# Comparison of clinicopathologic findings and urine drug screen results in cannabis-positive and control dogs

**DOI:** 10.3389/fvets.2025.1679400

**Published:** 2025-12-08

**Authors:** Helder Camilo da Silva Pereira, Wesley Marinho Brandão Barbosa, Mariana Ferreira Venceslau, Ricardo Romão Guerra, Lucas Rannier Ribeiro Antonino Carvalho

**Affiliations:** 1 Department of Veterinary Science, IESP Centro Universitário, João Pessoa, Brazil; 2 Department of Veterinary Medicine, UNIPÊ Centro Universitário de João Pessoa, João Pessoa, Brazil; 3 Postgraduate Program in Animal Science (PPGCAn), Federal University of Paraíba, Areia, Brazil; 4 Department of Physiology and Pharmacology, Karolinska Institutet, Stockholm, Sweden

**Keywords:** cannabis intoxication, dogs, THC metabolites, veterinary toxicology, endocannabinoid system

## Abstract

**Introduction:**

Cannabis intoxication is increasingly reported in small animal practice, often resulting from accidental ingestion of human products or unsupervised access to the plant. Although clinical signs are well documented, laboratory alterations remain poorly described. This study aimed to confirm cannabis intoxication in dogs using a rapid urinary test for THC metabolites and to investigate associated hematological, biochemical, and urinary alterations.

**Methods:**

In this study, nine dogs that were admitted to the emergency veterinary clinic in João Pessoa, Brazil, between 2021 and 2022, with suspected cannabis ingestion and owner confirmation, were evaluated. The animals were included regardless of breed, sex or age, and the results compared with healthy animals (control). Clinical signs were treated supportively. Urine and blood samples were collected 6 h after admission. Dogs were classified as “positive” or “negative/control” based on chromatographic detection of THC metabolites (PoCT COC/THC Assure Tech, Westlake Eco Zone/China; ANVISA 80885650015). Hematological analysis evaluated anemia, leukocyte, and platelet counts; serum biochemistry focused on hepatic and renal markers; urinalysis assessed color, clarity, specific gravity, and pH.

**Results:**

No statistically significant differences were observed between intoxicated and non-intoxicated dogs in any of the laboratory parameters. Additionally, most animals showed only discrete or absent clinical signs.

**Discussion:**

These findings suggest that accidental cannabis ingestion in dogs tends to result in mild physiological effects and minimal laboratory alterations, indicating a low risk of severe toxicity. Nonetheless, accurate identification of exposure using diagnostic tools is essential for appropriate case management. A deeper understanding of the endocannabinoid system in companion animals is key to improving clinical decision-making and assessing potential risks associated with cannabis exposure.

## Introduction

Intoxications are commonly observed in small animal clinical practice (1). These events often result from the accidental ingestion of toxic plants, the consumption of foods inappropriate for the species, or the incorrect administration of medications without veterinary guidance (2). *Cannabis* is a shrub native to Asia, belonging to the family Cannabaceae. It contains variable amounts of flavonoids, terpenes, and cannabinoids, with the latter being the main intoxicating active compounds when ingested beyond the animal's metabolic capacity (3).

In recent years, *Cannabis* sp. has emerged as a relevant toxic agent in domestic environments, particularly in regions where its recreational or medicinal use has been legalized. A study conducted in the United States evaluated trends in marijuana toxicosis in dogs between 2005 and 2010 in a state with legalized medical cannabis, reporting a fourfold increase in cases during the study period, totaling more than 120 reports (4). In Colombia (2023–2024), 113 cases of marijuana intoxication in dogs were documented, representing approximately 12% of all veterinary toxicology cases. In Brazil, a previous study reported that 3.3% of recorded intoxications cases in dogs were associated with cannabis ingestion (5). These findings highlight the growing relevance of cannabis intoxication in veterinary practice and emphasize the need for prompt diagnosis and treatment.

Because of their exploratory behavior, dogs are the species most frequently affected by accidental cannabis ingestion (6, 19, 20). In such cases, clinical management is improved when the causative agent is confirmed (7). Dogs intoxicated with cannabis may exhibit a variety of clinical signs, most commonly lethargy, ataxia, urinary incontinence, hypersalivation, and mydriasis (4), as well as vomiting (8). Other reported symptoms include disorientation, vocalization and bradycardia. However, these changes are generally mild and inconsistent among cases. This variability in both clinical presentation and laboratory results underscores the importance of combining clinical evaluation with confirmatory diagnostic tests—such as the rapid detection of urinary THC metabolites—to ensure accurate diagnosis and appropriate therapeutic decision-making (4).

While confirming the identity of the ingested agent in suspected cases on cannabis intoxication in dogs can provide certainty for clients, laboratory confirmation is not typically required for an adequate diagnosis to be made, nor will it alter clinical decision-making or treatment strategies. Laboratory confirmation of marijuana intoxication is often costly and time-consuming, requiring specialized expertise and equipment (9). However, the use of point-of-care (POC) tests has become increasingly feasible due to their low cost, ease of use, and rapid turnaround time. These tests enable timely clinical decisions and allow veterinarians to implement targeted therapeutic approaches (4).

The present study aimed to detect the presence of *Cannabis* metabolites in the urine of dogs using a rapid diagnostic test designed for the detection of human THC metabolites and to compare hematological, clinical biochemical, and urinalysis findings between cannabis-positive and control (cannabis-negative) dogs, as well as with established laboratory reference values. This approach facilitates a better understanding of the physiological effects of accidental cannabis ingestion in dogs and evaluates the diagnostic utility of human rapid urinary THC metabolite tests in veterinary emergency situations.

## Methods

This was a prospective observational study conducted in João Pessoa, in the Northeast region of Brazil, which analyzed cases of accidental ingestion of cannabis-derived products by dogs. Due to the exploratory nature of the investigation, no exclusion criteria were established regarding breed, age, or sex. The analysis included dogs admitted to the emergency service with a history of marijuana ingestion and clinical signs suggestive of cannabis intoxication, as well as cases in which owners reported possible ingestion of Cannabis or its derivatives.

Initially, an anamnesis was performed with the animal owners. During the interview, the main complaint was recorded, along with the estimated time since suspected cannabis ingestion, the product/compound and the clinical signs observed by the owner. Clinical management was tailored to each patient's general condition. Regardless of the source of intoxication, biological samples were collected for hematological and urinary analyses at two time points. First, upon admission, blood samples were obtained for hematological and biochemical analyses to assess the systemic impact of intoxication. Second, after clinical stabilization, urine samples were collected via ultrasound-guided cystocentesis approximately 6 h after hospitalization for THC metabolite testing.

Urinalysis included assessment of volume, color, appearance, specific gravity, and pH. Complete blood counts and serum biochemical analyses were also performed to evaluate renal, hepatic, and metabolic biomarkers; electrolytes were not measured.

Vital parameters, including temperature and blood glucose, were monitored, along with clinical signs such as vomiting, diarrhea, and dehydration, which were corrected as needed using conventional protocols. The dose of marijuana ingestion could not be estimated due to imprecision in the reported amount consumed by the animal, and at the time of the study, no laboratory in the state was equipped to quantify cannabis-derived compounds, which is a limitation of this work.

All patients were hospitalized, and a rapid urine test for detection of THC metabolites was performed approximately 6 h after hospital admission. Facing of the emergency nature of the cases, admission times were not standardized, and the interval prior to testing was estimated at an average of 6 h to align with the operational routine of the veterinary clinic.

Urine samples were collected by ultrasound-guided cystocentesis and immediately tested using the PoCT COC/THC Assure Tech (Westlake Eco Zone, Hangzhou, China; ANVISA Registration MS 80885650015, Minas Gerais, Brazil). This urine drug test is validated for human urine, and according to the manufacturer, the main urinary metabolite of marijuana is 11-nor-Δ^9^-tetrahydrocannabinol-9-carboxylic acid (THC-COOH) and its glucuronide conjugate in humans. However, other metabolites may also produce a positive reaction when present at the concentrations listed in Table 1, indicating possible cross-reactivity. The evaluation of other urinary parameters was performed using Labtest UriAction 10 colorimetric strips, ref. 122/100 (Labtest Diagnóstica S.A., Brazil).

**Table 1:** THC-related compounds that react to the Assure Tech PoCT COC/THC test.

**THC-related compounds**	**Concentration (ng/ml)**
11-nor-Δ^9^-tetrahydrocannabinol-9-carboxylic acid	50
11-nor-Δ^8^-tetrahydrocannabinol-9-carboxylic acid	50
Δ^9^-tetrahydrocannabinol	15,000
Δ^9^-tetrahydrocannabinol	15,000
Cannabinol	20,000

All data were tabulated and analyzed using GraphPad Prism 9 software. Based on the PoCT results, dogs were classified as “*cannabis-positive*” or “control (*cannabis-negative*).” Data distribution was assessed using the Shapiro–Wilk normality test. For variables showing a normal distribution, comparisons between groups were performed using the paired Student's *t*-test, whereas non-normally distributed data were analyzed using the Mann–Whitney *U* test. Differences were considered statistically significant at *p* ≤ 0.05. Results are expressed as mean ± standard deviation and were compared with established laboratory reference values.

## Results

During the study period, nine dogs were admitted to the veterinary emergency department presenting with clinical signs consistent with cannabis intoxication and a history of accidental ingestion reported by their owners. All nine tested positive for THC metabolites using the referenced point-of-care (PoC) test. As a control group, urine samples from nine healthy dogs—whose owners denied any exposure to toxic agents—were analyzed, and all tested negative.

Among the nine dogs testing positive for urinary THC metabolites, owners were asked during anamnesis about potential sources of intoxication. In most confirmed cases (seven), the primary route of exposure was the ingestion of marijuana cigarette remnants. Two cases differed in etiology: one involved the ingestion of butter infused with THC extract, and the other involved direct ingestion of a cannabis-containing cake.

All animals included in the study were admitted to the clinic during nighttime hours and were hospitalized for supportive medical care. Hospitalization not only allowed monitoring of the progression of intoxication but was also necessary to accommodate the clinic's daytime operational schedule and the start of laboratory and imaging services the following day.

**Figure 1:**
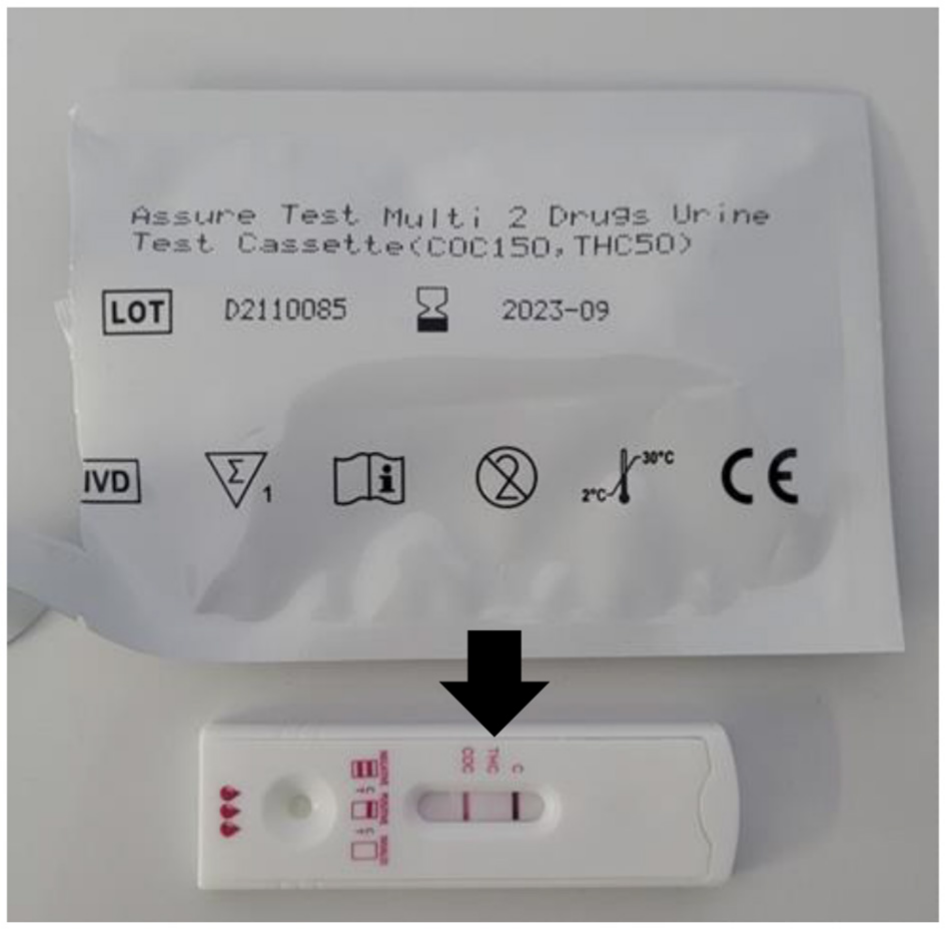
Chromatographic test for the detection of THC metabolites showing a positive result (arrow). PocT COC/THC Assure Tech, Westlake Eco Zone/China (Registered with ANVISA/MS 80885650015 – Minas Gerais, Brazil).

For the test procedure, a small volume of urine was applied to the reaction area of the test cassette. A positive result was indicated by the complete absence of coloration in the specific test zone for THC metabolites, as illustrated in Figure 1.

The clinical parameters of the patients evaluated in this study are presented in Table 2. The clinical signs observed were generally mild and subtle, characterized mainly by slight apathy and mild reluctance to move, with or without vomiting or diarrhea, and variable changes in body temperature and glycemia. No consistent pattern of abnormalities could be identified among the affected animals. Heart and respiratory rates remained within normal limits or showed only minor variations. All transient clinical alterations were promptly corrected with supportive care.

**Table 2:** Clinical parameters of dogs classified as cannabis-positive, or control (cannabis-negative) based on the PoCT COC/THC Assure Tech (Westlake Eco Zone, China; ANVISA/MS registration 80885650015 – Minas Gerais, Brazil).

**Parameters**	**PocT Positive (*n* = 9)**	**PocT Negative (*n* = 9)**	**Reference (18)**
Body temperature (°C)	38.5 ± 0.5	38.2 ± 0.5	37.5–39.1
Heart rate (bpm)	115.5 ± 13.3	113.7 ± 18.1	60–160
Respiratory rate (mpm)	26.6 ± 10.0	30.0 ± 11.4	10–40
Capillary refill time (s)	2 (2)^*^	2 (2)^*^	2
Dehydration^a^	2 (0–3)^*^	1 (0–3)^*^	1–3

Data are presented as mean ± standard deviation. ^a^Dehydration was clinically estimated according to Jericó et al. (18), based on percentage of body water loss and assigned a numerical score as follows: mild ≈5% = 1; moderate = 6–8% = 2; severe = 10–12% = 3; none = 0.

^*^Data are presented as median (range).

Hematological analyses were performed within a maximum interval of 12 h after blood collection. The results are presented in Table 3. No significant differences were observed when comparing the “positive” and “negative” groups. Furthermore, no alterations indicative of anemia, infection, inflammation, anaphylactic reaction, or platelet abnormalities were detected when compared to reference values.

**Table 3:** Hematological parameters of dogs classified as cannabis-positive, or control (cannabis-negative) based on the PoCT COC/THC Assure Tech (Westlake Eco Zone, China; ANVISA/MS registration 80885650015 – Minas Gerais, Brazil).

**Parameters**	**PocT Positive (*n* = 9)**	**PocT Negative (*n* = 9)**	**Reference (18)**
Erythrocytes (10^6^/μL)	7.0 ± 0.4	6.5 ± 2.4	5.5–8.5
Hematocrit (%)	45.0 ± 3.5	37.8 ± 10.0	37–55
Hemoglobin (g/dl)	15.0 ± 1.3	12.3 ± 3.2	12–18
Mean corpuscular volume (fl)	64.3 ± 3.0	61.7 ± 10.5	60–77
Mean corpuscular hemoglobin concentration (%)	33.3 ± 2.4	32.4 ± 1.2	31–36
Leukocytes (×10^9^/L)	17.2 ± 1.5	26.2 ± 22.1	6–17
Segmented neutrophils (×10^9^/L)	13.3 ± 2.0	15.3 ± 11.3	3–11
Band neutrophils (×10^9^/L)	0 ± 0	0 ± 0	0–500
Lymphocytes (×10^9^/L)	2.3 ± 1.2	3.4 ± 2.7	1–5
Eosinophils (×10^9^/L)	0.21 ± 0.35	0.13 ± 0.20	1–12
Monocytes (×10^9^/L)	0.14 ± 0.14	0.05 ± 0.05	1–13
Basophils (×10^9^/L)	0 ± 0	0 ± 0	rare
Platelets (×10^9^/L)	377.6 ± 214.0	249.0 ± 216.3	200–500
Total plasma proteins (g/dl)	7.2 ± 1.1	7.1 ± 1.3	6–8

Data are presented as mean ± standard deviation.

Urinalysis and the diagnostic test for intoxication were performed immediately after urine sample collection. The urinary parameters are presented in Table 4. Similar to the hematological evaluation, no notable urinary alterations were observed.

**Table 4:** Urinary parameters of dogs classified as cannabis-positive, or control (cannabis-negative) based on the PoCT COC/THC Assure Tech (Westlake Eco Zone, China; ANVISA/MS registration 80885650015 – Minas Gerais, Brazil).

**Parameters**	**PocT Positive (*n* = 9)**	**PocT Negative (*n* = 9)**	**Reference (18)**
Volume (ml)	10.2 ± 0.6	10.0 ± 0.5	–
Color^a^	2 (1–3)^*^	2 (1–3)^*^	1
Aspect^b^	2 (1–3)^*^	2 (1–3)^*^	1
Density	1.034 ± 15.2	1.031 ± 15.6	1.015–1.045
Ph	7.5 (6–8)^*^	7.0 (6.5–8)^*^	7–9

Data are presented as mean ± standard deviation. ^a^Color: light yellow = 1, dark yellow = 2, and brown = 3.

^b^Aspect: clear = 1, slightly turbid = 2, and turbid = 3 (18).

^*^Data are presented as median (range).

Regarding clinical biochemical analyses, serum measurements of creatinine, urea, alanine aminotransferase (ALT), alkaline phosphatase (ALP), and glucose were performed. The data are presented in Table 5. No significant alterations were observed in the evaluated parameters that would indicate renal or hepatic impairment, nor were there changes in glycemia suggestive of acute disturbances in glucose metabolism.

**Table 5:** Biochemical parameters of dogs classified as cannabis-positive, or control (cannabis-negative) based on the PoCT COC/THC Assure Tech (Westlake Eco Zone, China; ANVISA/MS registration 80885650015 – Minas Gerais, Brazil).

**Parameters**	**PocT positive (*n* = 9)**	**PocT Negative (*n* = 9)**	***Paired t test***	**Reference (18)**
Creatinine (mg/dl)	1.2 ± 0.3	1.6 ± 0.4	ns *p* = 0.3060	0.5–1.8
Urea (mg/dl)	49.6 ± 18.4	104.8 ± 76.1	ns *p* = 0.3107	12–54
Alanine Aminotransferase ALT (U/L)	59.3 ± 41.5	70.7 ± 28.7	ns *p* = 0.7623^*^	10–95
Alkaline phosphatase ALP (U/L)	119.4 ± 59.2	134.8 ± 84.7	ns *p* = 0.3564^*^	23–212
Blood glucose (mg/dl)	88.3 ± 14.1	89.9 ± 15.8	ns *p* = 0.8396	75–120

Data are presented as mean ± standard deviation. ^*^The observed values of ALT and ALP were non-normally distributed and were compared using the Mann–Whitney U test.

During treatment, non-specific clinical signs such as vomiting, diarrhea, and dehydration were managed supportively and resolved completely. Other clinical manifestations, including ataxia and behavioral alterations, were also monitored until full recovery. The duration of hospitalization ranged from 24 to 72 h, depending on the severity of clinical presentation. No animals died or were euthanized due to complications related to cannabis intoxication, as confirmed by clinical evaluation and/or point-of-care testing during the study period.

## Discussion

This study highlights the application of point-of-care (POC) testing for the rapid diagnosis of cannabis intoxication in dogs. The POC test proved to be a valuable tool for timely and accurate identification of the toxic agent, facilitating targeted clinical management (10). Therefore, in contrast to previous reports in the literature (4, 11), the human-based rapid test performed reliably in this veterinary setting.

Importantly, the comparison of laboratory results between cannabis-positive and negative animals revealed minimal physiological disturbances, suggesting that, although clinical signs may be present, cannabis intoxication in dogs does not typically result in significant hematological or biochemical alterations. This finding reinforces the notion that supportive care remains the cornerstone of treatment, while POC testing enhances diagnostic confidence and clinical decision-making.

The clinical alterations observed in this study were mild and consistent with those previously described in dogs exposed to cannabis. In the large retrospective cohort of 223 dogs with confirmed or suspected THC ingestion reported by Binagia et al. (8), the most frequent signs included neurological manifestations such as ataxia, lethargy, urinary incontinence, and hypersensitivity to stimuli, as well as cardiovascular and gastrointestinal alterations including tachycardia, bradycardia, vomiting, and hypersalivation. These clinical signs generally vary according to the amount and concentration of cannabinoids absorbed by the animal (8, 12, 13).

Regarding the routes of intoxication, oral ingestion was the exclusive exposure pathway identified in this study. The dogs ingested cannabis through various sources, including marijuana cigarette remnants, homemade foods containing cannabis, and butter infused with THC. These findings align closely with previous reports by Meola et al. (4), Miranda et al. (12), Amissah et al. (13), and Binagia et al. (8), which also identified oral consumption as the primary route of exposure in canine cannabis toxicosis. Oral ingestion is particularly concerning due to the high variable bioavailability and prolonged effects of cannabinoids when absorbed via the gastrointestinal tract, compared to inhalation. Furthermore, edible cannabis products pose an increased risk because they often contain concentrated amounts of cannabinoids and may be more appealing to pets due to their palatability. Understanding these common exposure routes is crucial for veterinary practitioners to accurately assess risk, provide appropriate client education, and tailor clinical interventions.

Similar to the hematological evaluation, the biochemical findings observed in this study appeared to reflect preexisting or non-specific conditions rather than direct effects of cannabis intoxication. No evidence of renal impairment was detected, which indicates that mild accidental exposures did not result in measurable kidney dysfunction. Although cannabinoids have been investigated for potential renal effects, our results do not support any protective or deleterious influence in this context. Likewise, hepatic enzyme values remained within reference limits, suggesting the absence of acute hepatic injury; however, previous studies have reported that cannabis or its derivatives may cause hepatocellular alterations under certain exposure conditions (14).

Regarding the clinical findings of temperature, heart rate, respiratory rate, etc., as well as any hematological changes observed in this study, they are likely related to pre-existing comorbidities, and not to cannabis intoxication itself. The literature does not report significant changes in red or white blood cell counts, nor platelet abnormalities, associated with cannabis intoxication in dogs. However, hematological analysis remains a valuable tool for assessing the patient's hydration status and overall physiological condition, allowing for more accurate correction of dehydration and supportive care tailored to individual needs.

In this study, the clinical findings related to hepatic evaluation are consistent with previous reports indicating that administration of relatively high doses of cannabinoids did not alter hepatic function in the animals studied (15). Regarding the unchanged levels of hepatic enzymes and glycemic index observed, other research has shown no significant changes in hepatic metabolites and even highlighted a positive effect of cannabis on insulin metabolism, demonstrating decreased insulin and triglyceride levels 30 min after ingestion of food or CBD, without impairing liver or kidney function (16).

Regarding urinalysis, no significant alterations related to cannabis intoxication were identified in the parameters of the urine samples. For this study, the most critical parameter was the presence or absence of cannabinoid compounds in the urine, for which complementary evaluation was necessary.

The chromatographic urine test used in this study is a lateral flow immunochromatographic test designed for the qualitative detection of tetrahydrocannabinol (THC) and its metabolites in human urine, with a cutoff concentration of 50 ng/ml for THC. The method is based on competitive antigen–antibody binding, in which the presence of THC or its metabolites above the cutoff inhibits the appearance of the test line. The primary metabolite detected by this test is THC-COOH. In veterinary context, unlike in humans, the production of THC-COOH has not been clearly demonstrated in dogs, and experimental and pharmacokinetic evidence indicates that canine metabolism favors the formation of 8-hydroxy-Δ^9^-THC (8-OH-THC) and 11-hydroxy-Δ^9^-THC (11-OH-THC) as the predominant metabolites (4, 11, 17). Therefore, the positive results obtained in this study likely reflect cross-reactivity of the assay with one or more of these canine metabolites rather than direct detection of THC-COOH.

Another relevant point for discussion is the interval between admission and testing. In this study, urine samples were analyzed approximately 6 h after clinical presentation, rather than immediately. This interval may have influenced the results, as most urinary immunoassays for cannabinoids detect the metabolite THC-COOH about 4 h after ingestion (8). Therefore, delayed testing likely increased the urinary concentration of detectable metabolites, thereby enhancing the apparent sensitivity of the assay without necessarily reflecting superior analytical performance. The manufacturer's instructions do not specify the ideal interval between exposure and testing; the recommendation is that the assay must be performed promptly after urine sample collection.

With respect to the limitations of this study, the relatively small sample size, inherent to its observational nature, reduces the statistical power to detect mild physiological variations previously associated with THC intoxication, such as subtle fluctuations in heart rate, respiratory rate, or body temperature. The absence of additional physiological parameters, including blood pressure, blood gas analysis, and electrolyte monitoring, also limited the evaluation of systemic responses that could have provided further insight into the cardiovascular and metabolic effects of THC in dogs. Moreover, confirmatory analytical testing using liquid chromatography–mass spectrometry (LC–MS) was not available, precluding definitive identification of the metabolites responsible for positive immunoassay results. Future studies with larger populations, comprehensive physiological monitoring, and validated confirmatory methods are warranted to better elucidate the metabolic profile of THC in dogs and to establish the diagnostic accuracy of point-of-care urine testing in veterinary settings.

## Conclusions

In this study, the point-of-care chromatographic urine test (PoCT COC/THC Assure Tech, Westlake Eco Zone/China) correctly identified all nine dogs with accidental cannabis ingestion and yielded negative results in control animals. Hematological, biochemical, and urinalysis parameters showed no significant differences between groups, suggesting that mild accidental exposures may not cause detectable systemic alterations. Although the sample size was limited, the findings indicate that this urine drug screen may offer greater sensitivity than those previously evaluated in veterinary medicine. Further studies with larger populations are warranted to confirm its diagnostic performance and clinical applicability in veterinary practice.

## Data Availability

The original contributions presented in the study are included in the article/supplementary material, further inquiries can be directed to the corresponding author.
